# SEX AND ASA CLASSIFICATION, NOT FASTING TIME, ARE ASSOCIATED WITH THE LIKELIHOOD OF COMPLICATIONS IN THE POSTOPERATIVE PERIOD

**DOI:** 10.1590/0102-6720202400027e1820

**Published:** 2024-10-28

**Authors:** Adeline Mariano Silva RESENDE, José Luis Braga de AQUINO, Vania Aparecida LEANDRO-MERHI

**Affiliations:** 1 Pontifícia Universidade Católica de Campinas, Graduate Program in Health Sciences – Campinas (SP), Brazil.

**Keywords:** Postoperative Complications, Nutritional Status, Fasting, Risk Factors, Complicações Pós-Operatórias, Estado Nutricional, Jejum, Fatores de Risco

## Abstract

**BACKGROUND:**

According to the literature, some factors are associated with the development of postoperative complications including surgical approach, smoking, comorbidities, nutritional status, classification of the American Society of Anesthesiologists (ASA), fasting time period, and others. In the case of surgical patients, some factors are important for the assessment of the outcomes.

**AIMS:**

To investigate the factors associated with the likelihood of postoperative complications in surgical patients.

**METHODS:**

A prospective observational study was conducted with patients who were admitted to hospital more than 24 h. The following variables were investigated: nutritional risk screening, body mass index, ASA classification, fasting time, length of hospital stay, and postoperative complications. For statistical analysis, the Chi-square, Fisher’s exact, and Mann-Whitney tests were used. To investigate the risk factors associated with postoperative complications, simple and multiple Cox regression analyses were used.

**RESULTS:**

In the total group of patients, there was an association between postoperative complications and men (p=0.0197), surgical risk (ASA) (p=0.0397) and length of hospital stay (p<0001); men showed a risk 2.2 times greater than women for some kind of postoperative complication (p=0.0456; PR=2.167; 95%CI 1.015–4.624). In patients undergoing gastrointestinal surgery, there was an association between postoperative complications and length of hospital stay (p<0001). In patients undergoing other surgeries, there was an association between postoperative complications and length of hospital stay (p<0001) and ASA classification (p=0.0160); ASA classification was considered a factor associated with the probability of postoperative complications (p=0.0335; PR=4.125; 95%CI 1.117–15.237).

**CONCLUSIONS:**

Men in the total group of patients and the ASA 3 or 4 criteria in the group of patients undergoing other surgeries were considered factors associated with the occurrence of complications in the postoperative period.

## RESUMO

**RACIONAL:**

A literatura tem mostrado que alguns fatores estão associados ao desenvolvimento de complicações pós-operatórias incluindo abordagem cirúrgica, tabagismo, comorbidades, estado nutricional, classificação da Sociedade Americana de Anestesiologistas (ASA), tempo de jejum e outros fatores. No caso de pacientes cirúrgicos, alguns fatores são importantes para a avaliação dos resultados.

**OBJETIVOS:**

Investigar os fatores associados à probabilidade de complicações pós-operatórias em pacientes cirúrgicos.

**MÉTODOS:**

Foi realizado um estudo observacional prospectivo com pacientes que ficaram internados por mais de 24 horas. Foram investigadas as seguintes variáveis: triagem de risco nutricional, índice de massa corporal, classificação ASA, tempo de jejum, tempo de internação e complicações pós-operatórias. Para análise estatística foram utilizados os testes Qui-quadrado, Exato de Fisher e Mann-Whitney. Para investigar os fatores de risco associados às complicações pós-operatórias foram utilizadas análises de regressão de Cox simples e múltipla.

**RESULTADOS:**

No grupo total de pacientes houve associação entre complicações pós-operatórias e sexo masculino (p=0,0197), risco cirúrgico (ASA) (p=0,0397) e tempo de internação (p<0001) e os homens apresentaram risco 2.2 vezes maior do que as mulheres de sofrer algum tipo de complicação pós-operatória (p=0.0456; RP=2.167; IC95% 1.015–4.624). Nos pacientes submetidos a cirurgias gastrointestinais houve associação entre complicações pós-operatórias e tempo de internação (p<0001). Nos pacientes submetidos a outras cirurgias houve associação entre complicações pós-operatórias e tempo de internação (p<0001) e classificação ASA (p=0,0160); e a classificação ASA foi considerada fator associado à probabilidade de complicações pós-operatórias (p=0,0335; RP=4,125; IC95% 1,117–15,237).

**CONCLUSÕES:**

O sexo masculino no grupo total de pacientes e o critério ASA 3 ou 4 no grupo de pacientes submetidos a outras cirurgias são fatores associados à presença de complicações no período pós-operatório.

## INTRODUCTION

Some factors associated with the development of postoperative complications had already been addressed by the relevant literature, including surgical approach, smoking, comorbidities, nutritional status, classification of the American Society of Anesthesiologists (ASA), fasting time period, and others^17,27,30,31,35^. Some risk factors have recently been reported in colorectal resection patients such as complex surgical procedures, postoperative albumin infusion, perioperative surgical events, and others^22^. In the case of surgical patients, some factors are important for the assessment of the outcomes, in such a way that it is crucial to understand these factors. Among them, for example, reducing length of hospital stay is one of the objectives of the enhanced recovery after surgery (ERAS) protocol^25,41^. Early postoperative ambulation and distant tumor metastases were also reported as protective factors^22^. Other reports in the literature have already demonstrated that the application of perioperative measures can achieve rapid postoperative recovery, reduce length of hospital stay, and reduce hospital costs^9,14,38^.

The guidelines based on the Acceleration of Total Postoperative Recovery (ACERTO) project and supported by evidence associated with perioperative nutritional care in elective General Surgery procedures, indicate that the ACERTO recommendations can accelerate the postoperative recovery of patients undergoing elective general surgery, with a reduction in morbidity, length of stay and readmission and, consequently, costs^1,2,6,10,11^. Hence, the application of multimodal protocols to support clinical practices in the perioperative assessment and the investigation of the factors that may influence the surgical outcomes, justify carrying out this investigation.

The objective of this study is to investigatethe factors associated with the likelihood of postoperative complications in surgical patients.

## METHODS

### Study design and location

This is a prospective observational cohort study, conducted in a university hospital with patients admitted under regular follow-up in clinical-surgical wards and intensive care units. For this study, the Strengthening the Reporting of Observational Studies in Epidemiology (STROBE) guidelines were followed^39^.

This study was approved by the institution’s Research Ethics Committee (number 5.973.659). All participants in this investigation received detailed information about the study and signed the Informed Consent Form. All procedures were carried out in accordance with the relevant guidelines and regulations.

### Participants, inclusion and exclusion criteria and sample size

The study inclusion criteria were: adult patients, with indication and submitted to surgery during hospital stay. Patients under the age of 18 years were excluded as well as those with incomplete data in their medical records and those who had to suspend the surgical procedure. After reviewing the inclusion and exclusion criteria, a population of 154 patients of both sexes, who were on hospital follow-up in the perioperative period, were considered eligible to participate in the study, based on the calculation of surgical procedures performed in the year prior to the research.

### Data collection and methodological procedures

Data were collected in the pre-and postoperative periods, initially surveying demographic data and then other variables. Data were collected directly from the patients’ medical records and during attendance of the inpatients who were under regular follow-up, by the actual study investigator.

### Study variables

a) Nutritional Risk Screening (NRS-2002): to assess nutritional risk, the Nutritional Risk Screening (NRS-2002) was used; this instrument has been internationally recommended for the early detection of nutritional risk in hospitalized patients and, in particular, in surgical patients^19,20,29^. This instrument allows rating nutritional risk by a numerical score, namely=3 (with nutritional risk) and <3 (without nutritional risk) scores^19,20^, after reviewing weight loss, body mass index, reduction in food intake, and disease severity.

b) Body mass index (BMI): body weight and height data were collected to determine the body mass index, according to the criteria standardized by the World Health Organization^42^ for adult patients up to 60 years of age (less than 18.5kg/m^2^=below the normal weight; 18.5–24.9 kg/m^2^=normal weight; 25.0–29.9 kg/m^2^=overweight; 30.0 kg/m^2^=obesity). For patients over 60 years of age, the cutoff points recommended by Lipschitz^24^ were considered (<22.0kg/m^2^=thinness; 22.0-27.0 kg/m^2^=adequate weight;>27.0 kg/m^2^=excess weight).

c) American Society of Anesthesiologists (ASA) Classification: to investigate the patient’s profile during the perioperative period, the ASA classification was considered (ASA I: normal health; ASA II: mild systemic disease; ASA III: severe non-disabling systemic disease; ASA IV: severe systemic disease, disabling, with serious threat to life; ASA V: moribund patient, with minimal survival expectation; ASA VI: brain-dead, organ donor patient)^13^. In this study, patients with classification up to ASA IV were included.

d) Fasting time indicated (prescribed) preoperatively: to evaluate the fasting time indicated in the preoperative period, the medical prescription for fasting was reviewed, following the standard description used in the hospital institution, that is, fasting after 10 pm or fasting after 12 am (midnight).

e) Total fasting time: to investigate the total preoperative fasting time, the time from the start of fasting to the point in time of the surgical procedure initiation was calculated, comparing the total fasting time with the recommendations of multimodal protocols (ACERTO)^1,6,10,11^.

f) Fasting abbreviation: to evaluate the abbreviation of fasting, the prescription of a carbohydrate-rich component (CHO) during preoperative fasting was investigated, as recommended by multimodal protocols (ACERTO)^1,6,10,11^.

g) Complications in the postoperative period: to investigate the evolution of patients in the postoperative period, the occurrence of clinical complications in the postoperative period was evaluated such as surgical wound (evaluation of the surgical incision healing process), gastrointestinal signs (weight loss and vomiting in the postoperative period), infectious signs (presence of abscesses and changes in body temperature), cardiovascular changes (bleeding, edema and blood pressure changes), and length of hospital stay (days)^4,32,33^.

### Statistical analysis

An initial descriptive analysis was carried out to characterize the population under study; frequency tables for the categorical variables, with absolute frequency (n) and percentage (%) values and for the quantitative variables were developed and descriptive measures were obtained (mean, standard deviation, and median). Subsequently, to compare proportions between two groups, the χ^2^ test or Fisher’s exact test were used, when necessary; for the comparison of continuous or orderable measurements, the Mann-Whitney test was employed.

Subsequently, to evaluate the factors associated with the presence of complications in the postoperative period, the univariate and multiple Cox regression analyses were used, with the Stepwise variable selection process. The level of significance adopted for all statistical tests was 5% (p<0.05). Data were reviewed using the computer program SAS System for Windows^8,34,40^.

### RESULTS

In this investigation, a total of 154 patients (n=154) were evaluated; they had been admitted for regular follow-up for surgical procedures in clinical-surgical wards and intensive care units, during the period between September 2022 and April 2023. In Table 1 we illustrate the characteristics of the assessed population, observing that 57.1% (n=88) of the investigation participants were men; out of the total investigation participants, 47.4% (n=73) underwent gastrointestinal tract surgery; 44.2% (n=68) were classified as ASA 2 and 42.2% (n=65), as ASA 3. Nutritional risk according to the NRS-2002 was assessed in 66.4% (n=77) of patients (Table 1). For analysis purposes, fasting time was stratified into >8h and >10h. Considering this classification, the total fasting time >8h was performed by 96.8% (n=149) patients and >10h by 85.1% (n=131). Regarding the abbreviation of fasting, 98.1% (n=153) did not ingest a carbohydrate-rich component during preoperative fasting (Table 1). We verified that 22.7% (n=35) of patients presented postoperative complications, most of them being cardiovascular (57.1%; n=20) (Table 1).

**Table 1:** Characteristics of the population assessed (n=154).

Variables	Classification	n (154)	%
Sex	Women	66	42.9
	Men	88	57.1
Surgical risk	ASA 1	17	10.9
	ASA 2	68	44.2
	ASA 3	65	42.2
	ASA 4	4	2.6
Type of surgery	Gastrointestinal	73	47.4
	Urological	21	13.6
	Orthopedic	20	13
	Neurological	14	9.1
	Vascular	14	9.1
	Thoracic	12	7.8
Nutritional Risk Screening (NRS-2002)	At risk	77	66.4
	Without risk	39	33.6
Fasting time (8 h)	=8 h	5	3.2
	>8 h	149	96.8
Fasting time (10 h)	=10 h	23	14.9
	>10 h	131	85.1
Fasting Abbreviation (CHO feeding)	No	151	98.1
	Yes	3	1.9
Postoperative complications	No	119	77.3
	Yes	35	22.7
Complication	Cardiovascular	20	57.1
	Gastrointestinal	14	40.0
	Infectious	14	40.0

ASA: American Society of Anesthesiologists; CHO: carbohydrate-rich component.

The average age of the investigation participants was 57.74 years (±17.72; median=62.72 years), body weight 73.85 kg (±16.61; median=72 kg); BMI 26.80 kg/m^2^ (±5.99; median=26.04 kg/m2); fasting time 18.32 hours (±9.23; median=15.58 hours); and hospitalization time 11.82 days (±14.54; median=7 days).

In Table 2 we compared the study variables with the occurrence or not of postoperative complications. In this analysis, we found statistical significance (p<0.05) for the variable sex (p=0.0197), with a higher proportion for men; patients with surgical risk (ASA) (p=0.0397) were classified into two groups: ASA 1 and 2 (healthy patients or with mild systemic disease) and ASA 3 and 4 (patients with severe, life-threatening systemic disease) and length of hospitalization (p<0001). We observed that patients who presented complications were hospitalized for a period longer than 21.49 days (±17.58). We found no significant differences in the other comparisons of the reviewed variables (Table 2).

**Table 2:** Descriptive analysis of the study variables and comparison with the presence or absence of postoperative complications.

Variables	Category	Postoperative complications		n	p-value
		No (%)	Yes (%)		
Age (years)		n=119	n=35	n=154	0.4102*
	Mean±SD	57.15±18.19	59.73±16.11	57.60±17.69	
	Median	62.73	62.72	62.72	
BMI (kg/m^2^)		n=112	n=35	n=147	0.5067*
	Mean±SD	27.01±5.97	26.57±6.13	26.90±5.99	
	Median	26.23	25.35	26.04	
Total fasting (h)		n=119	n=35	n=154	0.7140*
	Mean±SD	18.47±9.24	17.83±9.21	18.32±9.23	
	Median	15.67	15.50	15.58	
Sex		n=119	n=35	n=154	0.0197^†^
	Women	57 (47.9)	9 (25.7)	66 (42.9)	
	Men	62 (52.1)	26 (74.3)	88 (57.1)	
Fasting time=8 h		n=119	n=35	n=154	0.3187^‡^
	Yes	3 (2.5)	2 (5.7)	5 (3.2)	
	No	116 (97.5)	33 (94.3)	149 (96.8)	
Fasting time=10 h		n=119	n=35	n=154	0.3389^†^
	Yes	16 (13.4)	7 (20)	23 (14.9)	
	No	103 (86.6)	28 (80)	131 (85.1)	
NRS-2002		n=83	n=33	n=116	0.3615^†^
	At risk	53 (63.9)	24 (72.7)	77 (66.4)	
	Without risk	30 (36.1)	9 (27.3)	39 (33.6)	
Surgical risk		n=119	n=35	n=154	0.0397^†^
	ASA 1 and 2	71 (59.7)	14 (40)	85 (55.2)	
	ASA 3 and 4	48 (40.3)	21 (60)	69 (44.8)	
Type of surgery		n=119	n=35	n=154	0.0688^‡^
	GIT	50 (42)	23 (65.7)	73 (47.4)	
	URO	19 (16)	2 (5.7)	21 (13.6)	
	ORTOP	17 (14.3)	3 (8.6)	20 (13)	
	NEURO	13 (10.9)	1 (2.9)	14 (9.1)	
	VASC	9 (7.6)	5 (14.3)	14 (9.1)	
	TORAX	11 (9.2)	1 (2.9)	12 (7.8)	
Length of hospital stay (days)		n=119	n=35	n=154	<0.0001*
	Mean±SD	8.98±12.22	21.49±17.58	11.82±14.54	
	Median	6.00	15.00	7.00	

*Mann-Whitney Test;

^†^χ^2^ test;

^‡^Fisher’s exact test. SD: standard deviation; BMI: body mass index; ASA: American Society of Anesthesiologists; NRS-2002: Nutritional Risk Screening; GIT: gastrointestinal tract; URO: urological; ORTOP: orthopedic; NEURO: neurological; VASC: vascular; TORAX: thoracic.

In the comparison between all the study variables (age, sex, BMI, NRS-2002, type of surgery, length of hospital stay, and surgical risk-ASA) and preoperative fasting time equal to or greater than 8 h and equal to or greater than 10h, we found no statistical difference when expanding the analysis to prolonged fasting. In the comparison between the study variables and the patients’ sex, we observed a statistical difference only for the length of hospital stay (p=0.0195) variable. The other variables did not present statistically significant differences.

In Table 3 we show the factors associated with complications in the postoperative period, investigated by simple and multiple Cox regression analysis. We verified that only sex remained a factor associated with complications, with men having a 2.2 times higher risk than women of suffering some type of complication in the postoperative period (p=0.0456; PR=2.167; 95%CI 1.015–4.624).

**Table 3:** Study on factors associated with postoperative complications using simple and multiple Cox regression analysis.

Variables	Category	p-value	PR	95%CI	
Cox simple analysis Sex Age Body mass index Fasting time
	Men x Women	0.0456	2.167	1.015	4.624
		0.5056	1.007	0.987	1.027
		0.7404	0.990	0.935	1.049
		0.7504	0.994	0.958	1.032
ASA	(1 and 2) x (3 and 4)	0.0750	1.849	0.940	3.635
NRS-2002	At risk x Without risk	0.4420	1.351	0.628	2.905
Type of surgery	ORTOP x NEURO	0.5205	2.100	0.218	20.188
	GIT x NEURO	0.1463	4.411	0.596	32.662
	TORAX x NEURO	0.9132	1.167	0.073	18.652
	URO x NEURO	0.8143	1.333	0.121	14.704
	VASC x NEURO	0.1418	5.000	0.584	42.797
Fasting time 10 h	=10h x >10h	0.4030	0.702	0.307	1.608
Multiple analysis* Sex
	Men x Women	0.0456	2.167	1.015	4.624

*Modeling the probability of the presence of complications. PR: percentile rank; CI: confidence interval; ASA: American Society of Anesthesiologists; NRS-2002: Nutritional Risk Screening; ORTOP: orthopedic; NEURO: neurological; GIT: gastrointestinal tract; TORAX: thoracic; URO: urological; VASC: vascular.

Subsequently, the study variables were analyzed and compared with the type of surgery (gastrointestinal tract-GIT surgeries and other surgeries) to which the patients had been submitted. There was a statistical difference in relation to age (p=0.0297), postoperative complications (p=0.0136), and fasting time >10 h (p=0.0264). We found no significant difference in the other study variables in relation to the type of surgery. In the comparison between the study variables and the classified fasting time (=10 h and >10 h) in the two groups of surgeries (GIT surgeries and other surgeries), no statistical difference was evident.

When comparing the reviewed variables and the presence of complications in the postoperative period in the group of patients undergoing GIT surgeries, we found a statistical difference only for length of hospital stay (p<0001). The patients who presented complications in the postoperative period were those who remained hospitalized for the longest time (in patients who underwent GIT surgeries). In the comparison between the reviewed variables and the presence of complications in the postoperative period, in the group of patients undergoing other surgeries, we verified a statistical difference for the length of hospital stay (p<0001) and the ASA classification (p=0.0160). Patients who presented complications in the postoperative period were those who remained hospitalized for the longest time and who had been classified as ASA 3 and 4 (these are patients who underwent other surgeries).

In Tables 4 and 5 we illustrate the factors associated with postoperative complications investigated by simple and multiple Cox regression analysis, in patients undergoing GIT surgeries and other surgeries. There was no association between the variables in the group of patients undergoing GIT surgeries and no variable was significant at the 5% level (Table 4).

**Table 4:** Study on factors associated with postoperative complications, in the group of patients undergoing gastrointestinal tract surgeries, using simple and multiple Cox regression analysis (modeling the probability of presence of complications).

Variables	Category	p-value	PR	95%CI	
Cox simple analysis Sex Age Body mass index Fasting time
	Men x Women	0.1618	1.885	0.776	4.582
		0.7578	1.004	0.979	1.029
		0.6328	0.982	0.912	1.057
		0.8689	0.996	0.950	1.045
ASA	(3 and 4) x (1 and 2)	0.5912	1.251	0.552	2.836
NRS-2002	At risk x Without risk	0.8280	1.109	0.437	2.812
Fasting time 10h	=10 h x >10 h	0.4048	0.597	0.177	2.009
Multiple analysis* No variables significant at the 5% level were selected (n=71)					

*Stepwise variable selection process. PR: percentile rank; CI: confidence interval; ASA: American Society of Anesthesiologists; NRS-2002: Nutritional Risk Screening. The NRS variable was excluded from the multiple analysis due to the reduced number of patients in this analysis.

**Table 5:** Study on factors associated with postoperative complications in the group of patients undergoing other surgeries, using simple and multiple Cox regression analysis (modeling the probability of presence of complications).

Variables	Category	p-value	PR	95%CI	
Cox simple analysis Sex Age Body mass index Fasting time
	Men x Women	0.1109	3.437	0.753	15.686
		0.8801	1.002	0.971	1.035
		0.8919	1.006	0.918	1.103
		0.5809	0.981	0.918	1.049
ASA	(3 and 4) x (1 and 2)	0.0396	3.943	1.067	14.564
NRS-2002	At risk x Without risk	0.5071	1.580	0.409	6.111
Fasting time 10h	=10 h x >10 h	0.3016	0.531	0.160	1.764
Multiple analysis* ASA
	(3 and 4) x (1 and 2)	0.0335	4.125	1.117	15.237

*Stepwise variable selection process. PR: Percentile rank; CI: confidence interval; ASA: American Society of Anesthesiologists; NRS-2002: Nutritional Risk Screening. The NRS variable was excluded from the multiple analysis due to the reduced number of patients in this analysis.

In the group of patients undergoing other surgeries, the ASA classification was considered a factor associated with the probability of complications in the postoperative period; patients classified in categories 3 and 4 had a fourfold higher risk when compared to those classified in categories 1 and 2 (p=0.0335; PR=4.125; 95%CI 1.117–15.237) (Table 5).

**Figure 1:**
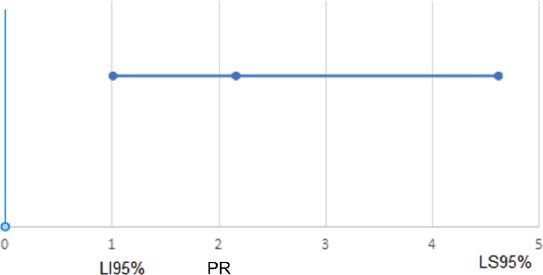
Percentile rank and respective 95% confidence interval (LI95%–LS95%) for men compared to women, estimated by Cox regression analysis, in the study on factors associated with the presence of complications in the postoperative period, considering the total group.

LI: Light interception; PR: percentile rank; LS: longitudinal study.

**Figure 2:**
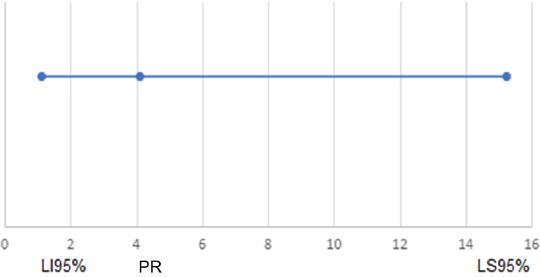
Percentile rank and respective 95% confidence interval (LI95%–LS95%) for American Society of Anesthesiologists criteria 3 or 4 compared to 1 or 2, estimated by Cox regression analysis, in the study of factors associated with the presence of complications in the postoperative period, considering the group of other surgeries. For the gastrointestinal tract surgery group, no variable was significant at the 5% level.

LI: Light interception; PR: percentile rank; LS: longitudinal study.

In Figures 1 and 2 we illustrate the percentile rank (PR) and respective 95% confidence interval (LI95–LS95%) for men, compared to women, and for ASA criteria 3 or 4, compared to criteria 1 or 2, estimated by the Cox regression analysis, in the assessment of factors associated with the presence of complications in the postoperative period, considering the total group and the group of other surgeries, respectively. For the GIT surgery group, no variable was significant at the 5% level.

## DISCUSSION

Most patients were men, with a profile similar to that described by Madan et al.^26^ and different from the profile of the population studied by Coeckelberghs et al.^7^, who evaluated 740 older adult patients, the majority of whom were women. The fasting time observed in the participants of this study was mostly prolonged, that is, the application of the intervention and time management did not follow the multimodal protocols^1,6,10,11^. Denkyi^12^, found that anesthesiologists in general have greater knowledge of preoperative fasting compared to other professionals and that the actual fasting time found in the evaluated patients was longer than the prescribed time; data similar to the findings of our study. GIT surgeries were prevalent in patients in our study; similar data were observed in a multicenter study conducted by Coeckelberghs et al.^7^, evaluating colectomy procedures.

The nutritional risk according to the NRS-2002 was verified in the majority of patients; these are relevant data of this investigation, although no statistical difference was observed in the comparison of nutritional risk between those who did or did not present complications in the postoperative period. Another relevant information was the identification of overweight patients, the majority of whom was measured by BMI. This discrepancy shows that the NRS instrument was more sensitive than BMI in identifying patients at nutritional risk. Masoudkabir et al.^28^, in a cohort study with patients with the same profile found in the present study, demonstrated that overweight and normal weight patients presented similar postoperative results, but patients with a BMI greater than 30 kg/m^2^ presented a significantly higher risk for mortality. Authors of another study, in which the perioperative risks associated with BMI were reviewed, showed that patients with a BMI=40 kg/m^2^ presented statistical significance for cardiac complications, and those with a BMI<18.5 kg/m^2^ had lower short- and long-term survival^3^.

In the present study, the majority of participants were classified as having an ASA 2 or ASA 3 risk, a similar profile found in the study conducted by Madan et al.^26^; observing that many patients had serious systemic diseases, requiring alertness for health care planning. A statistical difference was observed for surgical risk in patients classified under ASA 3 and 4; in other words the presence of serious systemic diseases may have resulted in postoperative complications. Madan et al.^26^, in a randomized study with ASA-1 and 2-profile patients undergoing coloproctological surgery, compared the application of the ERAS protocol, obtaining no statistical difference associated with postoperative complications. Thus, it is clear that patients with a more critical classification (ASA 3 and 4) may be at greater risk of postoperative complications; these data corroborate our findings.

A statistical difference was observed regarding sex, suggesting that postoperative complications could occur more frequently in men. Although there was no statistical difference, most of the complications found were related to the cardiovascular system (bleeding, edema, and changes in blood pressure levels). Another relevant finding was the association between prolonged length of hospital stay and the presence of postoperative complications. In the study conducted by Lindemann et al.^23^, comparing the application of the ERAS guidelines, the group submitted to this protocol (ERAS) presented a statistically significant reduction in the length of hospital stay. In our investigation, we observed no statistical difference related to preoperative fasting time; however, the study conducted by Lindemann et al.^23^, corroborates the findings of the present study with regard to the relation between the hospitalization period and the application of the ERAS guidelines, and may raise future hypotheses about the association between the length of hospitalization time and prolonged fasting. The preoperative period should be rigorously evaluated, considering noncompliance with these guidelines in the clinical practice despite the existence of current protocols; thus, this is a factor that could be modified to prevent complications^14,16,37^.

In a cohort study conducted by Sinha et al.^36^, the postoperative evolution of liquid and solid food intake, passage of flatus, catheter removal, ambulation, pain, and length of hospital stay in two groups of patients were investigated; all these variables occurred statistically less in the group that had undergone ERAS interventions, confirming one of the findings of this study in relation to length of hospital stay and its association with postoperative complications.

The findings of our investigation indicated that men were at greater risk than women for some type of complication in the postoperative period. The variables studied here were also compared among patients undergoing GIT surgeries and other surgeries; they indicated an association with older age, more postoperative complications, and fasting time >10 hours in patients with GIT surgeries. When comparing the variables with the occurrence of complications in the two groups of surgeries, patients hospitalized for longer periods presented more complications in the group of patients undergoing GIT surgeries. Those who remained hospitalized for longer periods and who had been classified as ASA 3 and 4 presented more complications in the group of patients undergoing other surgeries. Although we found no statistical difference between the variables in this study and the fasting time classified as 10 hours, we observed that, in both groups of surgeries, the majority of patients undergoing fasting time >10 hours presented nutritional risk according to the NRS, indicating the need for future studies to delve deeper into this issue.The difficulty in accessing the patients’ records in their entirety due to the fact that they were not fully computerized, especially in relation to complications, as well as the loss of some nutritional indicators, were the main limiting factors of this study.

Another finding was that, using regression analysis in the group of patients undergoing GIT surgeries, we found no association between the variables assessed in relation to complications. In the group of patients undergoing other surgeries, the ASA classification was considered a factor associated with the probability of complications in the postoperative period; patients classified in categories 3 and 4 presented a fourfold higher risk when compared to those classified in categories 1 and 2. In patients classified as ASA 3 and undergoing gastrointestinal, urogenital, or orthopedic surgeries, a similar profile as in our investigation was found; a prospective multicenter study conducted by Bartha et al.^5^, evaluated the ASA classification and surgical severity and showed that both classifications can be used to identify a high-risk surgical population in relation to postoperative morbidity and mortality for gastrointestinal and orthopedic surgery. The findings of the present investigation are in line with the findings reported by Bartha et al.^5^, with regard to the application of the ASA classification in the preoperative assessment; the results constitute a guidance for the prevention of perioperative complications. There are also reports of an association between malnutrition and postoperative complications and longer hospitalization periods in older adult patients, showing that advanced age and low BMI were considered risk factors for malnutrition^15^. As in patients undergoing sleeve gastrectomy, there was a significantly shorter length of hospital stay in the group of patients in which the ERAS protocol was applied and there was no increase in the rate of perioperative morbidity^21^. Other findings reported in the literature indicate a reduction of hospital stay in patients undergoing surgical procedures to whom the ERAS protocol had been applied^18,21,23^. Such reports suggest the application of multimodal protocols in surgical practice.

The hypothesis raised at the beginning of this study that preoperative fasting time could influence the occurrence of postoperative complications was not confirmed in our investigation. However, relevant findings were observed, such as a prolonged preoperative fasting period, nutritional risk according to the NRS in most patients, more postoperative complications in those patients with longer length of hospital stay, longer length of hospital stay among male patients, more general postoperative complications, greater occurrence of postoperative complications in male patients, in older age patients and fasting time >10 hours in patients undergoing gastrointestinal tract surgery, association between longer length of hospital stay and postoperative complications in patients with gastrointestinal tract surgery and other surgeries. Using the Cox regression, criteria 3 and 4 in the ASA classification were considered factors associated with the probability of postoperative complications in patients undergoing other surgeries. Considering the total group of patients, men were also considered a factor associated with the probability of postoperative complications.

## CONCLUSIONS

According to our findings, men in the total group of patients and the ASA 3 or 4 criteria in the group of patients undergoing other surgeries were considered factors associated with the occurrence of complications in the postoperative period.
